# Breakfast Consumption and Its Associations with Health-Related Behaviors among School-Aged Adolescents: A Cross-Sectional Study in Zhejiang Province, China

**DOI:** 10.3390/ijerph13080761

**Published:** 2016-07-27

**Authors:** Meng Wang, Jie-Ming Zhong, Hao Wang, Ming Zhao, Wei-Wei Gong, Jin Pan, Fang-Rong Fei, Hai-Bin Wu, Min Yu

**Affiliations:** Zhejiang Provincial Center for Disease Control and Prevention, 3399 Binsheng Road, Hangzhou 310051, China; mwang@cdc.zj.cn (M.W.); jmzhong@cdc.zj.cn (J.-M.Z.); hwang@cdc.zj.cn (H.W.); mzhao@cdc.zj.cn (M.Z.); wwgong@cdc.zj.cn (W.-W.G.); jpan@cdc.zj.cn (J.P.); frfei@cdc.zj.cn (F.-R.F.); hbwu@cdc.zj.cn (H.-B.W.)

**Keywords:** breakfast, adolescents, nutrition, behavior

## Abstract

Evidence indicates that breakfast consumption is associated with a cluster of health-related behaviors, yet studies in mainland China are scarce. This study is conducted to describe the frequency of breakfast consumption among Chinese adolescents and examine its associations with other dietary, physical activity, sedentary, sleep, cigarette-smoking, and alcohol-drinking behaviors. Breakfast consumption and other health-related behaviors data was collected via a self-administered questionnaire in a cross-sectional study in Zhejiang Province, China. A total of 19,542 school-aged adolescents were recruited in this survey. The associations between breakfast consumption and other health-related behaviors were examined using logistic regression models. A significantly higher prevalence of daily breakfast consumption was found among students who were younger (*p* for trend <0.001), from urban schools (*p* < 0.001), and academic high schools (*p* < 0.001). More frequent vegetable and milk consumption, greater physical activity, and longer sleep duration were positively associated with daily breakfast consumption, while soft drinks and fast food consumption, computer use, cigarette-smoking and alcohol-drinking behaviors were inversely associated. The prevalence of irregular breakfast consumption was relatively high among Chinese adolescents in Zhejiang Province. Daily breakfast consumption was associated with a constellation of health-related behaviors.

## 1. Introduction

Breakfast is widely considered to be the most important meal of the day. Considerable studies have revealed that consumption of a regular breakfast contributes to improving overall nutritional status in children and adolescents [1,2]. In addition, adolescents who consume breakfast have been shown to have better overall quality of life, school attendance and academic achievement [3,4,5,6]. Concomitantly, studies have linked skipping breakfast to adverse health outcomes of overweight and obesity, type 2 diabetes mellitus, and metabolic syndrome [7,8,9,10]. Despite the importance of breakfast consumption, a high prevalence of skipping breakfast among children and adolescents is widely reported in many countries across the world [11,12]. For Chinese populations, although the breakfast consumption habit is understudied, according to some certain surveys, not eating breakfast is also a public health concern, especially among adolescents and young adults [13,14]. 

Adolescence is a critical time of transition and lifestyle patterns develop during adolescence and continue through to adulthood. To formulate comprehensive health promotion strategies, a number of studies have explored the interrelations between multiple health-related behaviors, which involve dietary patterns, as well as smoking cigarettes, drinking alcohol, and physical activity [15,16,17,18]. Recently, the associations between breakfast consumption and a clustering of health-related behaviors have received more attention from public health professionals. However, to date, the relevant studies in mainland China are still lacking. On these grounds, the primary objective of this study is to (1) describe the frequency of breakfast consumption among school-aged adolescents in Zhejiang Province, China; (2) examine the associations between breakfast consumption and other dietary, physical activity, sedentary, sleep, cigarette-smoking, and alcohol-drinking behaviors.

## 2. Materials and Methods

### 2.1. Study Sample and Data Collection

In 2012, a school-based cross-sectional study on the risk behaviors of youth was conducted in the province of Zhejiang, China. The sampling procedures and sample characteristics are described in detail elsewhere [19] and are thus only briefly recounted here. When drawing the samples, a multistage, stratified cluster sampling technique was used. In the first stage, 30 counties, including 12 urban areas and 18 rural areas, were sampled from all 90 counties of Zhejiang Province on the basis of socioeconomic status. Then, all schools in selected counties were stratified according to their levels (middle school, academic and vocational high school) and geographical positions (from west to east, from north to south). In the second stage, based on the number of students in each level of school, samples of classes were chosen in each level of school. Finally, students in all selected classes were invited to participate in the survey. In total selected 12 urban areas and 18 rural areas, 20,589 middle and high school students from 490 classes (253 middle school classes, 122 academic high school classes and 115 vocational high school classes) agreed to participate in the survey, yielding a response rate of 97.53%. After excluding 108 ineligible questionnaires and 939 students who were not the natives of Zhejiang Province, finally, 19,542 students were recruited in the present study. A self-administered questionnaire was used to access the students’ dietary, as well as physical activity, sedentary, sleep, cigarette-smoking, and alcohol-drinking behaviors. Without teachers present, students filled in the anonymous questionnaire in the classroom independently. Once finished, questionnaires were collected on the spot by the researchers. To make all the participants voluntary, parents and school officials were sent a written letter to inform them that a study was to be conducted to examine issues relevant to adolescent health, and given the option to refuse the students’ participation in the study. Besides, all the researchers were strictly trained to protect the students’ privacy and ensure the confidentiality of the personal data. In particular, our study abided by the “Declaration of Helsinki” and was approved by the ethics committee of Zhejiang Provincial Center for Disease Control and Prevention (approval code: T-043-R).

### 2.2. The Definition of Breakfast Consumption and Other Behaviors

As the main interest of this study, breakfast consumption was accessed by the question: “During the past 30 days, in an average week, how many days did you eat breakfast?” with five answer options, “0 day”, “1–2 days”, “3–4 days”, “5–6 days”, “7 days”. The answers were further classified into three groups, “Never” (0 day), “sometimes” (one to two days, three to four days, and five to six days), and “daily” (seven days). Other behaviors included diet, physical activity, sedentarism, sleep, smoking cigarettes, and drinking alcohol are described in Table 1. Additionally, some explanatory variables were also taken into consideration in this study. They were age range (<13, 14, 15, >16 years), gender (girls, boys), location of school (urban, rural), school level (middle school, academic high school, vocational high school).

### 2.3. Statistical Analysis

Descriptive statistics were used to estimate the prevalence of breakfast consumption among school-aged adolescents. The associations between breakfast consumption, daily breakfast consumption and demographic characteristics of gender, age, location of school, and school level were tested with Chi-square tests. Notably, the linear trend of age was tested with linear-by-linear association Chi-square test. To explore the association of breakfast consumption and other health-related behaviors, logistic regression analyses were conducted by three steps in sequence. Firstly, univariate logistic regression analyses were carried out to preliminarily assess the association. Secondly, a multivariate logistic regression model was constructed by adding all the variables. Thirdly, another two multivariate logistic regression analyses were conducted to determine the effects of breakfast consumption on health-related behaviors in different gender and location of school. The effect values were reported by odds ratio (OR), adjusted odds ratio (AOR) with their 95% confidence intervals (CIs). A *p*-value of <0.05 was considered to be statistically significant. All analyses were performed using SAS statistical package (version 9.2, SAS Institute, Inc., Cary, NC, USA).

## 3. Results

### 3.1. Associations between Breakfast Consumption and Demographic Characteristics

Out of 19,542 students participating in the study, 7533 (38.55%) did not consume breakfast daily. The frequency of breakfast consumption in relation to demographic characteristics was shown in Table 2. Gender, age, location of school and school level were significantly associated with breakfast consumption. For daily breakfast consumption, the prevalence was significantly lower among older students compared to younger students (Chi-square value = 14.83, *p* for trend <0.001), higher among urban schools than rural schools (Chi-square value = 12.12, *p* < 0.001) and higher among academic high school students than vocational high school students (Chi-square value = 236.93, *p* < 0.001). There was no significant difference by gender for daily breakfast consumption (Chi-square value = 0.09, *p* = 0.769).

### 3.2. Associations between Breakfast Consumption and Health-Related Behaviors

Table 3 and Table 4 showed the associations between breakfast consumption and other health-related behaviors. Compared to those who were never breakfast consumers, daily breakfast consumers were 3.32 times (AOR = 3.32, 95% CI = 2.52–4.36) more likely to consume vegetables at least twice in a day and 2.06 times (AOR = 2.06, 95% CI = 1.55–2.72) more likely to consume milk at least three days in a week. These effects of sometimes eating breakfast were much weaker and the AORs were 2.43 (95% CI = 1.85–3.20) and 1.45 (95% CI = 1.10–1.92), respectively. Stratified by gender and the location of the school, these associations were much stronger for boys and urban school students. Daily breakfast consumption was significantly associated with the decreased probability of soft drink consumption at least once in a day (AOR = 0.59, 95% CI = 0.41–0.85) and fast food consumption at least two days in a week (AOR = 0.43, 95% CI = 0.31–0.60). These effects of daily breakfast consumption were particularly strong for girls (except for fast food consumption which was lower in boys) and rural school students. Breakfast consumption, whether daily or sometimes, was significantly associated with higher levels of physical activity (except for muscle strengthening activity). Daily breakfast consumers were 1.53 times (AOR = 1.53, 95% CI = 1.16–2.01) more likely to do moderate physical activity and 1.96 times (AOR = 1.96, 95% CI = 1.38–2.79) more likely to attend physical education classes compared to those who never consumed breakfast. Daily breakfast consumption was significantly associated with the decreased probability of computer use at least two hours in a day (AOR = 0.61, 95% CI = 0.46–0.81) and the effect was much stronger for girls and rural school students. Daily breakfast consumption was significantly associated with longer sleep duration as compared to those who never consume breakfast (AOR = 1.53, 95% CI = 1.15–2.04). Daily breakfast consumption was significantly associated with the decreased probability of cigarette smoking as compared to those who never consume breakfast (AOR = 0.39, 95% CI = 0.26–0.58). The result was similar for drinking alcohol (AOR = 0.66, 95% CI = 0.49–0.89), while significant associations were only for girls and rural school students.

## 4. Discussion

This study was conducted to examine the patterns of breakfast consumption among school-aged adolescents in Zhejiang Province, China, and its associations with health-related behaviors. To our knowledge, the present study was probably the first attempting to address the relationships between breakfast consumption and other health-related behaviors in mainland China. According to the current study, an important finding was that more than one third of these school students in Zhejiang Province consumed breakfast irregularly (<7 day/week). Although a direct comparison was impossible due to differences in the dietary habits, study subjects, and the definition of breakfast consumption, our result was similar to that found for primary school students in Hong Kong [13] and high school students in India [20]. Sex differences in regular breakfast consumption have been well studied, while the findings were inconclusive [11,21,22]. In our study, we did not find significant differences in the frequency of daily breakfast consumption between boys and girls. In the current study, older adolescents were found to consume breakfast irregularly more often, compared to those who are younger, which was consistent with previous studies [11,16,20]. The age disadvantage of older adolescents in breakfast consumption could be explained by the decreased parental influence, greater concerns about their body image and time considerations [16,23]. Several studies have explored the association between social economic status (SES) and breakfast consumption habits, which almost consistently suggested that the irregular breakfast habit was more common among adolescents of low SES [11,18]. In the current study, we used the area level of the school as an SES indictor and found that students belonging to rural schools were more likely to consume breakfast irregularly, which was in line with the previous studies. In our study, we also explored the frequency of breakfast consumption among students with different school levels, that was scarcely involved in previous studies. In this study, we found that the irregular breakfast consumption was more common in those students from vocational high schools and the reasons were uncertain. However, given that nutritional knowledge is important in developing dietary habits and is taught less in vocational high schools than in academic high schools, we proposed that the lower nutritional knowledge should be responsible for this finding.

In our study, the higher consumption of vegetables and milk and the lower consumption of soft drinks and fast food were found among daily or sometimes breakfast consumers as compared to those who never consume breakfast. These results were in line with earlier studies [20,24] and supported the finding that breakfast consumers tended to make healthier food choices throughout the day [25,26]. Although the association between breakfast consumption and physical activity was inconclusive [27,28,29], the present result suggested that students who ate breakfast sometimes or daily were more active than those who never did, especially for doing moderate physical activity and attending physical education classes. With regard to sedentary activity, our study found that students with daily breakfast consumption were less likely to use the computer more than two hours per day. However, similar research conducted in India did not find a significant association between breakfast consumption and computer use on weekdays or weekends [20]. Among the adolescents in this study, daily breakfast consumption was significantly associated with ≥8 h of sleep duration per day. The result was consistent with earlier studies conducted in other countries [30,31]. An inverse association between breakfast consumption and smoking and alcohol-drinking behavior has been documented in previous studies, respectively [16,17,18]. Our results confirmed these findings and also found that daily breakfast consumption was inversely associated with cigarette-smoking and alcohol-drinking behavior in Chinese adolescents. 

The results from our study were subjected to several limitations. First, with a cross-sectional design, we could not identify causal associations in the current study and further longitudinal research is needed. Second, the behaviors data relied on self-administered questionnaires and is likely to be susceptible to misreporting. Third, both breakfast consumption and other health-related behaviors could be also associated with obesity, which was not adjusted for in the multivariate analysis process. We would be glad to see if obesity mediates the associations between breakfast consumption and these health-related behaviors among Chinese adolescents in further research.

## 5. Conclusions 

In conclusion, the irregular breakfast consumption among Chinese adolescents in Zhejiang Province is a public health concern. Daily breakfast consumption was associated with healthy lifestyle patterns, such as better food choices, greater physical activity, longer sleep duration, and reduced behaviors of computer use, smoking cigarettes and drinking alcohol. To formulate health promotion programs and educational intervention strategies for the adolescence period, when lifestyle patterns are developing, more emphasis should be placed on regular breakfast consumption.

## Figures and Tables

**Table 1:** The definition of dietary, physical activity, sedentary, and other behaviors.

Behaviors	Definition
**Dietary**	
Fruits (≥2 times/day)	During the past 30 days, how many times did you eat fruits every day? (0 time, <1 time, 1 time, 2 times, 3 times, 4 times, ≥5 times)
Vegetables (≥2 times/day)	During the past 30 days, how many times did you eat vegetables every day? (0 time, <1 time, 1 time, 2 times, 3 times, 4 times, ≥5 times)
Milk (≥3 day/week)	During the past 30 days, how many days did you drink milk every week? (0 day, <1 day, 1–2 days, 3–4 days, 5–7 days)
Soft drinks (≥1 times/day)	During the past 7 days, how many times did you drink soft drinks, such as Coke, Pepsi, or Sprite? (0 time, 1–3 times, 4–6 times, once a day, twice a day, three times a day, ≥4 times a day)
Fast food (≥2 day /week)	During the past 7 days, how many days did you eat fast food, such as hamburger, hotdogs, or potato chips, etc.? (0 day, 1 day, 2 days, 3 days, 4 days, 5 days, 6 days, 7 days)
**Physical activity**	
Moderate physical activity (≥2 day/week)	During the past 7 days, how many days did you participate in at least 60 minutes of any kind of physical activity that increased your heart rate and made you breathe hard, such as kicking shuttlecock, fast bicycling, and doing housework, etc.? (0 day, 1 day, 2 days, 3 days, 4 days, 5 days, 6 days, 7 days)
Muscle strengthening activity (≥2 day/week)	During the past 7 days, how many days did you do exercises to strengthen or tone your muscles, such as push-ups, sit-ups, or weight lifting, etc.? (0 day, 1 day, 2 days, 3 days, 4 days, 5 days, 6 days, 7 days)
Attend physical education classes (≥2 day/week)	In an average week when you are in school, on how many days do you go to physical education classes? (0 day, 1 day, 2 days, 3 days, 4 days, 5 days, 6 days, 7 days)
**Sedentary Activity**	
Watch TV (≥2 h/day)	On an average school day, how many hours do you watch TV? (0 h, <1 h, 1–2 h, 2–3 h, 3–4 h, 4–5 h, ≥5 h)
Use computer (≥2 h/day)	During the past 7 days, in an average day, on how many hours did you spend using a computer for playing games, surfing, e-mailing, or watching movies, etc.? (open question)
**Other**	
Sleep duration (≥8 h/day)	During the past 30 days, how many hours did you spend on sleeping every day? (open question)
Smoke cigarettes (yes)	During the past 30 days, on how many days did you smoke cigarettes? (0 days, 1–2 days, 3–5 days, 6–9 days, 10–19 days, 20–29 days, 30 days)”. Participants who answered they had smoked at least 1 day during the past 30 days were considered to have the cigarette-smoking behavior
Drink alcohol (yes)	During the past 30 days, on how many days did you have at least one drink of alcohol? (0 days, 1–2 days, 3–5 days, 6–9 days, 10–19 days, 20–29 days, 30 days)”. Participants who answered they had drunk alcohol at least 1 day during the past 30 days were considered to have the alcohol-drinking behavior

**Table 2:** Demographic characteristics of students and the associations with breakfast consumption.

Characteristics	N (%)	Breakfast			Chi-Square	*p*	Chi-Sqaure *	*p* *
		Never (*n* = 232)	Sometimes (*n* = 7301)	Daily (*n* = 12,009)				
Gender					14.30	0.001	0.09	0.769
Boys	9723 (49.75)	144	3614	5965				
Girls	9819 (50.25)	88	3687	6044				
Age range (years)					57.37	<0.001	14.83 ^∆^	<0.001 ^∆^
≤13	3904 (19.98)	40	1299	2565				
14-	3038 (15.54)	45	1156	1837				
15-	3302 (16.90)	52	1346	1904				
≥16	9298 (47.58)	95	3500	5703				
Location of school					27.32	<0.001	12.12	<0.001
Urban	7346 (37.59)	112	2605	4629				
Rural	12,196 (62.41)	120	4696	7380				
School level					515.86	<0.001	236.93	<0.001
Middle school	9617 (49.21)	131	3505	5981				
Academic high school	5495 (28.12)	42	1748	3705				
Vocational high school	4430 (22.67)	59	2048	2323				

* Chi-square test for the associations of daily breakfast consumption and demographic characteristics; **^∆^** Linear-by-linear association chi-square test result.

**Table 3:** Breakfast consumption and its associations with health-related behaviors among all students.

Behaviors	Breakfast ^†^	All Students			
		OR (95% CI)	AOR (95% CI)		
**Dietary**					
Fruits (≥2 times/day) ^†^	Never	Ref.		Ref.	
	Sometimes	1.30 (0.97–1.74)		1.06 (0.78–1.45)	
	Daily	**1.59 (1.19–2.12)**		1.28 (0.94–1.75)	
Vegetables (≥2 times/day) ^†^	Never	Ref.		Ref.	
	Sometimes	**2.59 (1.98–3.40)**		**2.43 (1.85–3.20)**	
	Daily	**3.73 (2.85–4.87)**		**3.32 (2.52–4.36)**	
Milk (≥3 day/week) ^†^	Never	Ref.		Ref.	
	Sometimes	**1.45 (1.11–1.90)**		**1.45 (1.10–1.92)**	
	Daily	**2.26 (1.73–2.94)**		**2.06 (1.55–2.72)**	
Soft drinks (≥1 times/day) ^‡^	Never	Ref.		Ref.	
	Sometimes	**0.67 (0.47–0.94)**		0.84 (0.58–1.20)	
	Daily	**0.43 (0.31–0.61)**		**0.59 (0.41–0.85)**	
Fast food (≥2 day/week) ^‡^	Never	Ref.		Ref.	
	Sometimes	**0.63 (0.46–0.87)**		**0.65 (0.46–0.90)**	
	Daily	**0.39 (0.29–0.54)**		**0.43 (0.31–0.60)**	
**Physical Activity**					
Moderate physical activity (≥2 day/week) ^‡^	Never	Ref.		Ref.	
	Sometimes	**1.53 (1.18–1.99)**		**1.49 (1.13–1.96)**	
	Daily	**1.69 (1.30–2.19)**			**1.53 (1.16–2.01)**
Muscle strengthening activity (≥2 day/week) ^‡^	Never	Ref.			Ref.
	Sometimes	0.82 (0.62–1.07)			0.79 (0.59–1.07)
	Daily	0.99 (0.76–1.30)			0.91 (0.68–1.23)
Attend physical education classes (≥2 day/week)	Never	Ref.			Ref.
	Sometimes	**1.86 (1.36–2.54)**			**1.82 (1.28–2.59)**
	Daily	**2.43 (1.79–3.32)**			**1.96 (1.38–2.79)**
**Sedentary Activity**					
Watch TV (≥2 h/day)	Never	Ref.			Ref.
	Sometimes	1.05 (0.79–1.41)			1.01 (0.74–1.37)
	Daily	**0.70 (0.53–0.94)**			0.78 (0.58–1.06)
Use computer (≥2 h/day) ^‡^	Never	Ref.			Ref.
	Sometimes	0.90 (0.69–1.17)			0.95 (0.71–1.26)
	Daily	**0.49 (0.38–0.64)**			**0.61 (0.46–0.81)**
**Other**					
Sleep duration (≥8 h/day) ^†^	Never	Ref.			Ref.
	Sometimes	1.26 (0.96–1.65)			1.23 (0.93–1.64)
	Daily	**1.40 (1.07–1.83)**			**1.53 (1.15–2.04)**
Smoke cigarettes (yes) ^†^	Never	Ref.			Ref.
	Sometimes	**0.59 (0.42–0.82)**			0.77 (0.52–1.13)
	Daily	**0.23 (0.17–0.33)**			**0.39 (0.26–0.58)**
Drink alcohol (yes) ^†^	Never	Ref.			Ref.
	Sometimes	0.81 (0.62–1.06)			1.03 (0.76–1.39)
	Daily	**0.42 (0.32–0.55)**			**0.66 (0.49–0.89)**

Bold numbers represent significant results. ^†^ During the past 30 days; ^‡^ During the past seven days. OR: Odds ratio; AOR: Adjusted odds ratio; CI: Confidence interval; Ref: Reference group.

**Table 4:** Breakfast consumption and its associations with health-related behaviors stratified by gender and location of school.

Behaviors	Breakfast ^†^	Gender		Location of School	
		Boys	Girls	Urban	Rural
		AOR (95% CI)	AOR (95% CI)	AOR (95% CI)	AOR (95% CI)
**Dietary**					
Fruits (≥2 times/day) ^†^	Never	Ref.	Ref.	Ref.	Ref.
	Sometimes	0.97 (0.64–1.46)	1.09 (0.67–1.77)	0.86 (0.55–1.34)	1.28 (0.83–1.99)
	Daily	1.18 (0.79–1.78)	1.24 (0.76–2.01)	1.14 (0.73–1.76)	1.41 (0.91–2.19)
Vegetables (≥2 times/day) ^†^	Never	Ref.	Ref.	Ref.	Ref.
	Sometimes	**2.90 (2.01–4.19)**	**1.64 (1.05–2.56)**	**3.65 (2.38–5.60)**	**1.57 (1.07–2.29)**
	Daily	**3.95 (2.74–5.70)**	**2.09 (1.34–3.26)**	**4.90 (3.20–7.49)**	**2.03 (1.39–2.97)**
Milk (≥3 day/week) ^†^	Never	Ref.	Ref.	Ref.	Ref.
	Sometimes	**1.48 (1.03–2.12)**	1.12 (0.72–1.74)	1.42 (0.95–2.12)	1.29 (0.88–1.90)
	Daily	**2.33 (1.63–3.34)**	1.46 (0.94–2.27)	**2.16 (1.45–3.22)**	**1.79 (1.21–2.63)**
Soft drinks (≥1 times/day) ^‡^	Never	Ref.	Ref.	Ref.	Ref.
	Sometimes	1.04 (0.67–1.63)	**0.45 (0.24–0.82)**	0.83 (0.49–1.39)	0.66 (0.40–1.08)
	Daily	0.73 (0.47–1.14)	**0.32 (0.18–0.59)**	0.59 (0.35–1.00)	**0.47 (0.28–0.76)**
Fast food (≥2 day/week) ^‡^	Never	Ref.	Ref.	Ref.	Ref.
	Sometimes	**0.56 (0.37–0.86)**	0.70 (0.41–1.19)	0.81 (0.50–1.31)	**0.53 (0.33–0.84)**
	Daily	**0.41 (0.27–0.63)**	**0.42 (0.25–0.72)**	**0.57 (0.36–0.92)**	**0.34 (0.21–0.53)**
**Physical Activity**					
Moderate physical activity (≥2 day/week) ^‡^	Never	Ref.	Ref.	Ref.	Ref.
	Sometimes	1.44 (1.00–2.08)	1.57 (0.99–2.47)	1.29 (0.85–1.95)	**1.67 (1.13–2.47)**
	Daily	1.44 (1.00–2.07)	1.51 (0.96–2.38)	1.20 (0.80–1.82)	**1.68 (1.13–2.48)**
Muscle strengthening activity (≥2 day/week) ^‡^	Never	Ref.	Ref.	Ref.	Ref.
	Sometimes	0.85 (0.59–1.22)	0.66 (0.39–1.11)	**0.64 (0.42–0.97)**	0.72 (0.48–1.09)
	Daily	0.98 (0.68–1.40)	0.76 (0.45–1.27)	0.72 (0.47–1.10)	0.85 (0.56–1.27)
Attend physical education classes (≥2 day/week)	Never	Ref.	Ref.	Ref.	Ref.
	Sometimes	**2.39 (1.63–3.51)**	0.72 (0.37–1.43)	**1.61 (1.04–2.50)**	1.36 (0.82–2.27)
	Daily	**2.68 (1.83-3.94)**	0.83 (0.42–1.64)	**1.91 (1.23–2.95)**	1.56 (0.93–2.59)
**Sedentary Activity**					
Watch TV (≥2 h/day)	Never	Ref.	Ref.	Ref.	Ref.
	Sometimes	1.07 (0.72–1.59)	0.86 (0.53–1.39)	1.08 (0.67–1.74)	0.90 (0.60–1.34)
	Daily	0.80 (0.54–1.19)	0.63 (0.39–1.01)	0.82 (0.51–1.32)	0.66 (0.44–1.00)
Use computer (≥2 h/day) ^‡^	Never	Ref.	Ref.	Ref.	Ref.
	Sometimes	1.11 (0.78–1.59)	0.91 (0.58–1.43)	1.07 (0.72–1.61)	0.93 (0.63–1.38)
	Daily	**0.66 (0.46–0.93)**	**0.59 (0.37–0.92)**	**0.66 (0.44–0.99)**	**0.58 (0.39–0.85)**
**Other**					
Sleep duration (≥8 h/day) ^†^	Never	Ref.	Ref.	Ref.	Ref.
	Sometimes	1.08 (0.75–1.55)	1.32 (0.84–2.07)	1.05 (0.71–1.57)	1.21 (0.81–1.80)
	Daily	1.40 (0.98–2.01)	1.40 (0.90–2.20)	1.35 (0.91–2.00)	1.35 (0.90–2.01)
Smoke cigarettes (yes) ^†^	Never	Ref.	Ref.	Ref.	Ref.
	Sometimes	1.00 (0.64–1.55)	**0.30 (0.15–0.57)**	0.92 (0.51–1.65)	0.63 (0.38–1.06)
	Daily	**0.54 (0.34–0.84)**	**0.12 (0.06–0.23)**	**0.50 (0.28–0.90)**	**0.31 (0.19–0.53)**
Drink alcohol (yes) ^†^	Never	Ref.	Ref.	Ref.	Ref.
	Sometimes	1.36 (0.93–2.00)	**0.59 (0.36–0.96)**	1.55 (0.99–2.43)	0.70 (0.47–1.05)
	Daily	0.96 (0.65–1.41)	**0.34 (0.21–0.55)**	0.99 (0.63–1.56)	**0.45 (0.30–0.67)**

Bold numbers represent significant results. ^†^ During the past 30 days; ^‡^ During the past seven days. OR: Odds ratio; AOR: Adjusted odds ratio; CI: Confidence interval; Ref: Reference group.
